# Modeling the signaling endosome hypothesis: Why a drive to the nucleus is better than a (random) walk

**DOI:** 10.1186/1742-4682-2-43

**Published:** 2005-10-19

**Authors:** Charles L Howe

**Affiliations:** 1 Departments of Neuroscience and Neurology, Mayo Clinic College of Medicine, Guggenheim 442-C, 200 1^st ^Street SW, Rochester, MN 55905, USA

## Abstract

**Background:**

Information transfer from the plasma membrane to the nucleus is a universal cell biological property. Such information is generally encoded in the form of post-translationally modified protein messengers. Textbook signaling models typically depend upon the diffusion of molecular signals from the site of initiation at the plasma membrane to the site of effector function within the nucleus. However, such models fail to consider several critical constraints placed upon diffusion by the cellular milieu, including the likelihood of signal termination by dephosphorylation. In contrast, signaling associated with retrogradely transported membrane-bounded organelles such as endosomes provides a dephosphorylation-resistant mechanism for the vectorial transmission of molecular signals. We explore the relative efficiencies of signal diffusion versus retrograde transport of signaling endosomes.

**Results:**

Using large-scale Monte Carlo simulations of diffusing STAT-3 molecules coupled with probabilistic modeling of dephosphorylation kinetics we found that predicted theoretical measures of STAT-3 diffusion likely overestimate the effective range of this signal. Compared to the inherently nucleus-directed movement of retrogradely transported signaling endosomes, diffusion of STAT-3 becomes less efficient at information transfer in spatial domains greater than 200 nanometers from the plasma membrane.

**Conclusion:**

Our model suggests that cells might utilize two distinct information transmission paradigms: 1) fast local signaling via diffusion over spatial domains on the order of less than 200 nanometers; 2) long-distance signaling via information packets associated with the cytoskeletal transport apparatus. Our model supports previous observations suggesting that the signaling endosome hypothesis is a subset of a more general hypothesis that the most efficient mechanism for intracellular signaling-at-a-distance involves the association of signaling molecules with molecular motors that move along the cytoskeleton. Importantly, however, cytoskeletal association of membrane-bounded complexes containing ligand-occupied transmembrane receptors and downstream effector molecules provides the ability to regenerate signals at any point along the transmission path. We conclude that signaling endosomes provide unique information transmission properties relevant to all cell architectures, and we propose that the majority of relevant information transmitted from the plasma membrane to the nucleus will be found in association with organelles of endocytic origin.

## Background

**Figure 1:**
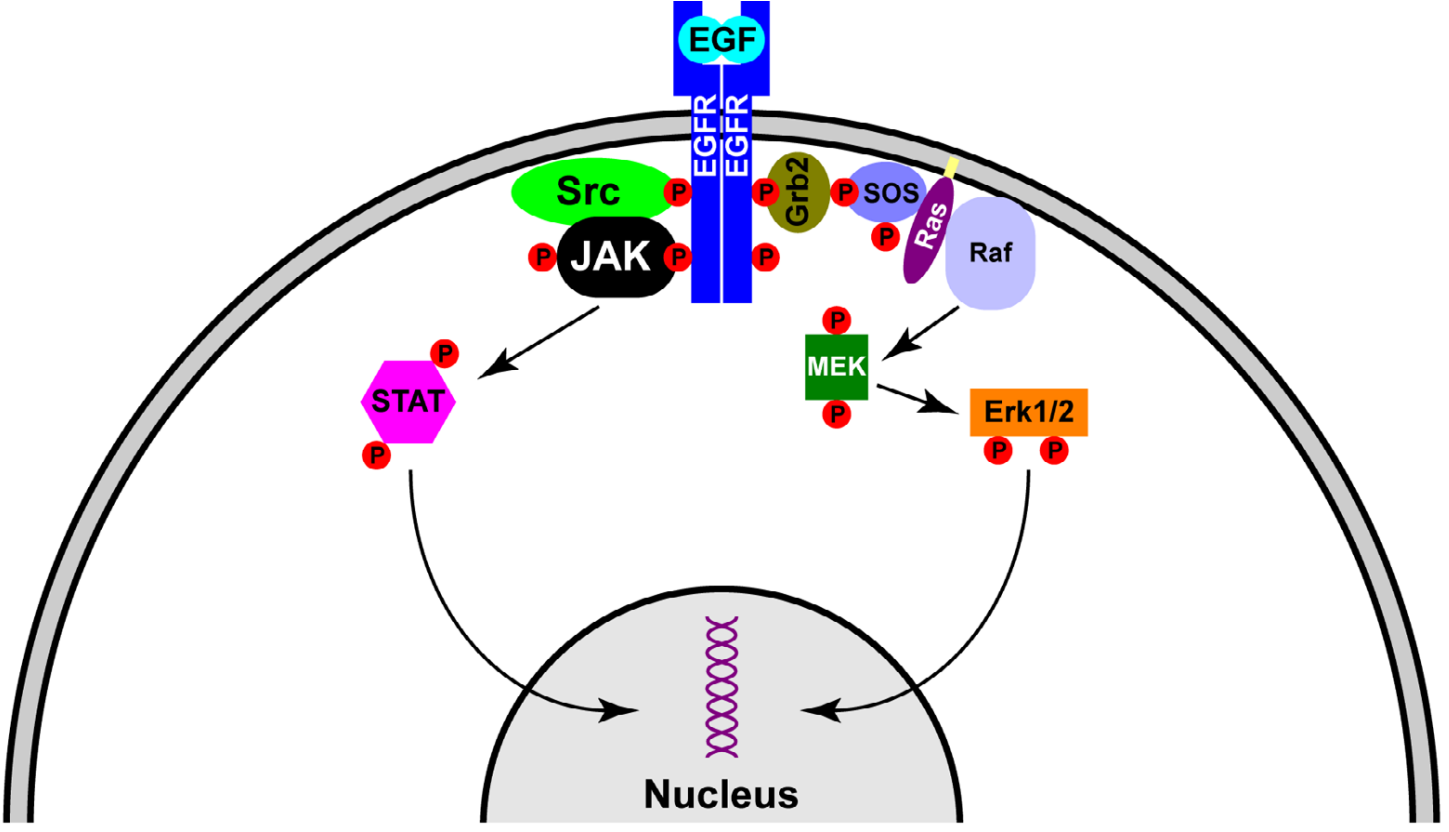
Simplified diagram showing the activation of STAT-3 and Erk1/2 downstream from EGF binding to EGFR. In the general model of signal transduction, the cascading chain of phosphorylation events culminating in activation of transcription factors such as STAT-3 and Erk1/2 depends upon the diffusion of these molecules from the site of signal initiation at the plasma membrane to the site of transcriptional regulation within the nucleus.

The transmission of signals from the extracellular surface of the plasma membrane to the nucleus is a complex process that involves a large repertoire of trafficking-related and signal-transducing proteins. A highly dynamic and carefully orchestrated series of molecular events has evolved to ensure that signals emanating from outside the cell are communicated to the nuclear transcriptional apparatus with fidelity and signal integrity. The classic model for the execution of this molecular symphony is a cascade of protein:protein interactions resulting in the spread of an amplified wave of protein phosphorylation that eventually culminates in a cadence of transcription factor activity. For example, as illustrated in Figure 1, epidermal growth factor (EGF) binds to it receptor tyrosine kinase (EGFR) on the surface of a cell, resulting in the transmission of a wave of tyrosine, serine, and threonine phosphorylation events that leads to the activation and nuclear translocation of several transcription factors, including STAT-3 (signal transducer and activator of transcription-3) and ERK1/2 (extracellular signal-related kinase-1/2; also known as mitogen-activated protein kinase, MAPK). This cascading wave model depends inherently upon the notion that activated transcription factors diffuse through the cytoplasm, enter the nucleus, and execute a program of transcriptional activation. Conceptually, this model is easy to grasp – but does it accurately reflect the biology and the physical constraints of cellular architecture? The answer appears to be "No", as a significant body of work over the past decades has challenged the fundamental validity of the diffusion model [1-3] and has offered elegant alternative models for the transmission of intracellular signals [4,5].

**Figure 2:**
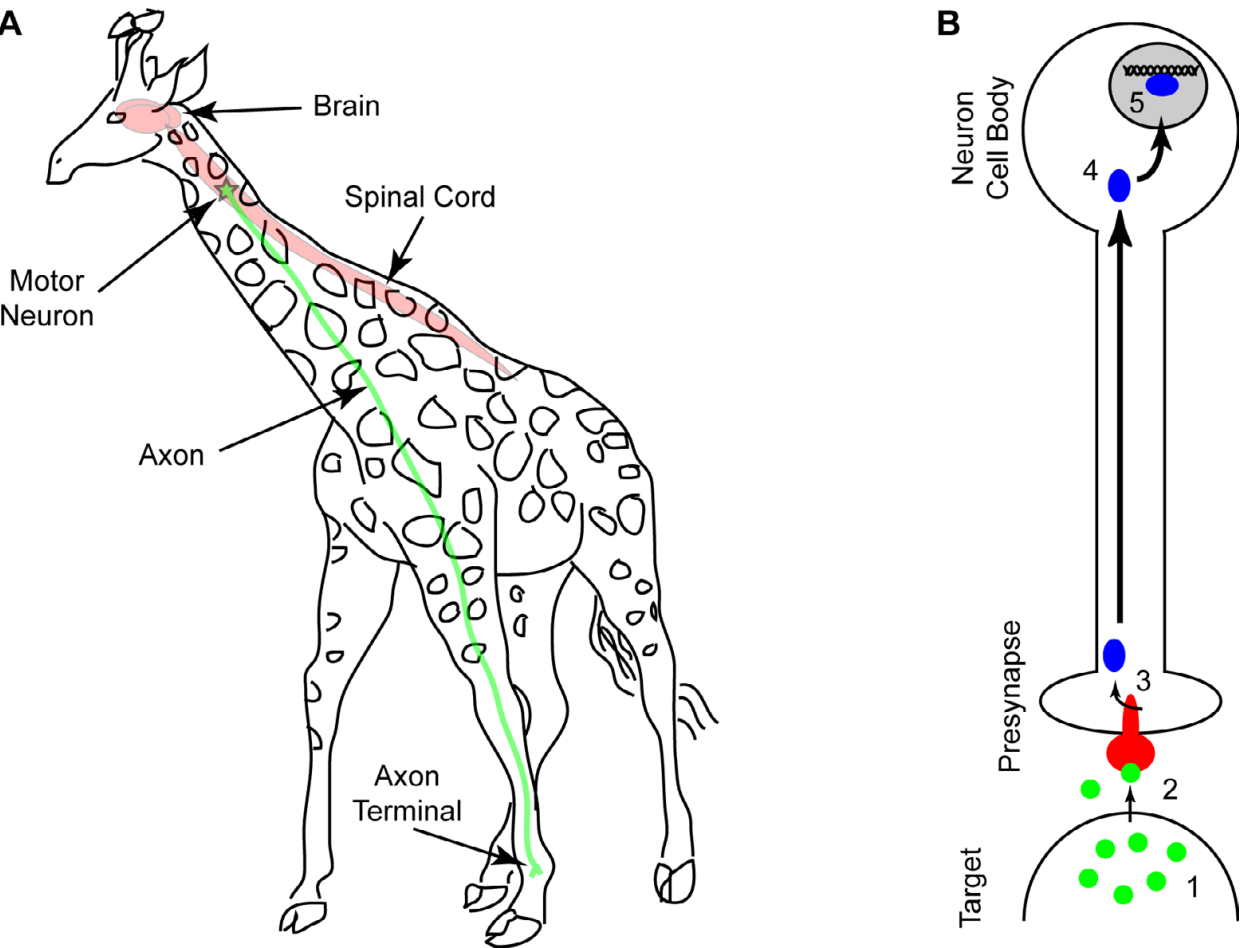
**A**) Neurons throughout the nervous system send axonal projections over distances ranging from microns to meters. For large or anatomically specialized animals such as the giraffe or the whale, more than 5 meters may separate the neuron cell body from the distal axon terminal. **B**) During development, neurons establish trophic interactions with target tissues. As an organism develops, the strength and maintenance of these trophic interactions determine whether neurons survive or die. Soluble protein trophic factors released by the target tissue (1) bind to transmembrane receptors on the presynaptic axon terminal (2), inducing receptor activation and the induction of intracellular signaling cascades (3). These signals must travel from the site of initiation to the distant cell body (4) in order to enter the nucleus and elicit transcriptional changes that determine the survival of the cell. This long-distance information transfer is a universal theme in neurodevelopment.

**Figure 3:**
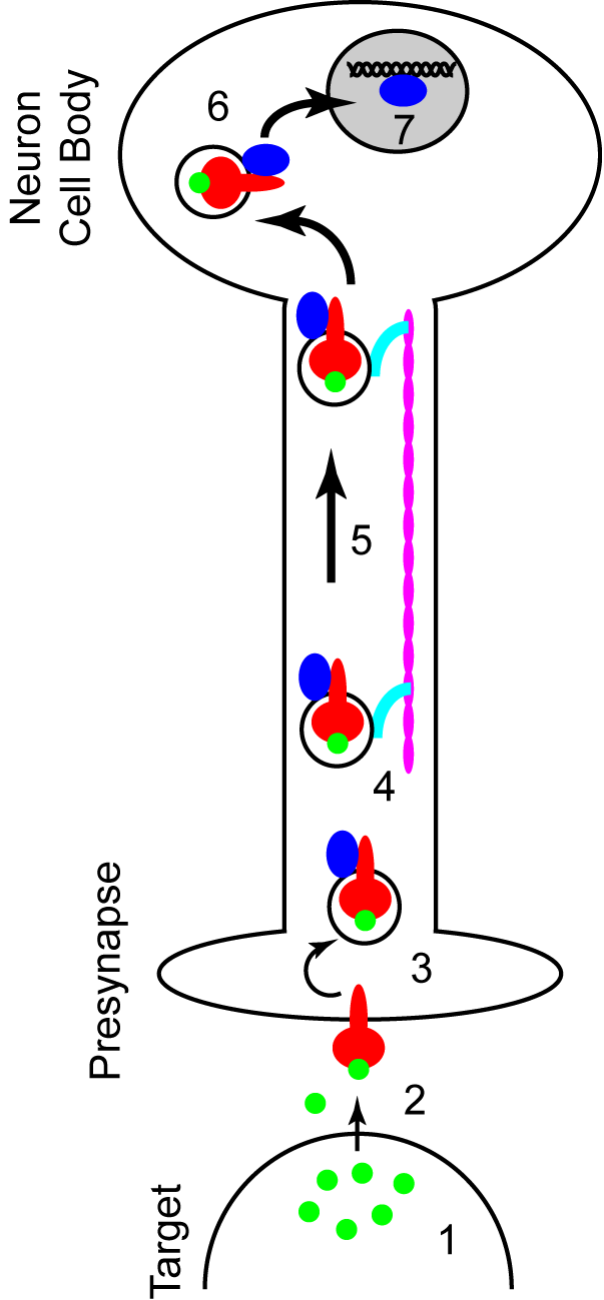
The signaling endosome hypothesis of long-distance axonal signal transmission. Soluble protein trophic factors released by the target (1) bind to transmembrane receptors on the presynaptic axon terminal (2), inducing receptor activation and internalization via clathrin-coated membranes or other endocytic structures (3). These endocytic vesicles give rise to transport endosomes that bear the receptor and associated signaling molecules as well as molecular motors (shown in turquoise) (4) that utilize microtubules (shown in pink) within the axon to carry the endosome toward the cell body (5). Upon arrival at the neuron cell body the endosome-associated signals may either initiate additional local signals or may directly translocate (6) into the nucleus to elicit transcriptional changes (7).

Neurons exhibit a unique architecture that places severe physical limitations on the possible mechanisms for translocation of signals. As shown in Figure 2A, projection neurons extend axons into target fields over distances that dwarf the dimensions of the cell body. And yet, the Neurotrophic Factor Hypothesis of neurodevelopment requires that target-derived soluble trophic factors induce signals in the presynaptic terminal of axons that result in transcriptional and translational changes in the nucleus and neuronal cell body (Figure 2B) [6]. While it is possible that a signal generated at the plasma membrane of the presynaptic terminal diffuses along the length of the axon in order to elicit an effect at the nucleus – it is not at all probable [5]. For some projection neurons the length of the axon is five orders of magnitude greater than the diameter of the neuron cell body, and the axoplasm therefore constitutes 1000-fold more volume than the cytoplasm of an average cell. The Signaling Endosome Hypothesis posits that an active, directed process of signal transmission is required to overcome the physical constraints of axonal distances and volumes [7]. Specifically, this hypothesis states that the most efficient mechanism for signaling-at-a-distance involves the packaging of a secreted growth factor signal into a discrete, coherent, membrane-bounded organelle that is moved along the length of the axon via a cytoskeleton-based transport machine (Figure 3) [7]. Indeed, a substantial body of research supports the signaling endosome hypothesis within the context of neurotrophin signaling in neurons [8-12]. However, while the unique geometry of neurons provides a teleological basis for the existence of signaling endosomes, it is far more interesting to posit that the signaling endosome hypothesis represents a general biological mechanism for signal transduction and signal compartmentalization [4]. Such a generalized hypothesis might state that the most efficient mechanism for communicating signals from the plasma membrane to the nucleus is the compartmentalization of signal transducers into quantal endocytic membrane-associated signaling packets that are retrogradely transported along microtubules through the cytoplasm. By utilizing the intrinsic directionality and nucleus-directed organization of the cellular microtubule network, signaling endosomes provide a noise-resistant mechanism for the vectorial transport of plasma membrane-derived signals to the nucleus.

A number of findings support the concept that signaling from internal cellular membranes is a general phenomenon that is relevant to understanding receptor tyrosine kinase signaling in many cellular systems. For example, EGFR, as discussed above, is internalized via clathrin-coated vesicles following EGF-binding and receptor activation [13-15]. In the past, trafficking through this compartment was considered part of a normal degradative process that removes activated receptors from the plasma membrane and thereby truncates and controls downstream signaling [16]. But while this certainly remains a critical function of endocytosis, recent experiments demonstrate that EGFR remains phosphorylated and active following internalization [17], and that downstream signaling partners such as Ras colocalize with these internalized, endosome-associated receptors [18-23]. Moreover, the signals emanating from these internalized EGFR are biologically meaningful, as cell survival is directly supported by such signaling [24]. Likewise, Bild and colleagues recently observed that STAT-3 signaling initiated by EGFR activation localized to endocytic vesicles that moved from the plasma membrane to the nucleus, and they found that inhibition of EGFR endocytosis prevented STAT-3 nuclear translocation and abrogated STAT-3-mediated gene transcription [25]. However, while evidence supports the existence of signaling endosomes, it does not rule out simultaneous diffusion-based signal transduction.

**Figure 4:**
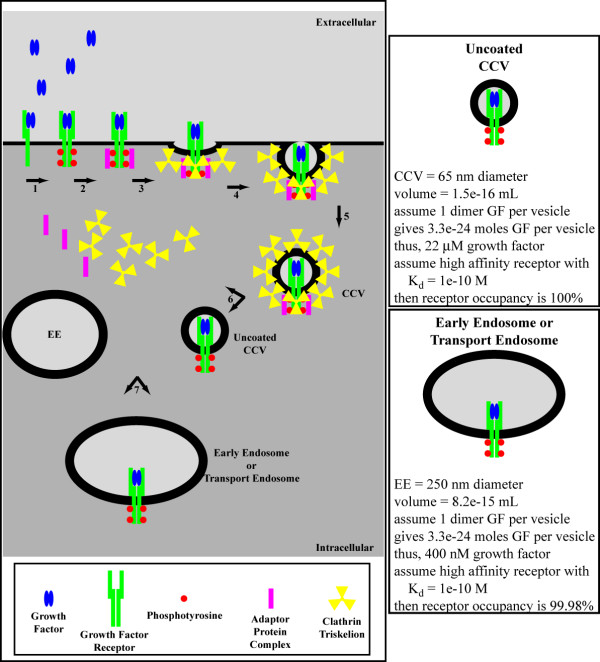
Growth factor receptors are internalized into clathrin-coated vesicles (CCVs) following ligand binding and receptor activation (1–5). These CCVs are uncoated (6) and mature into early endosomes (EE) (7) that may serve as transport endosomes [48]. The concentration of growth factor in transport endosomes is high enough to guarantee effectively 100% receptor occupancy. Hence, if the endosome-associated receptor encounters a phosphatase, the phosphorylation signal is rapidly regenerated.

**Figure 5:**
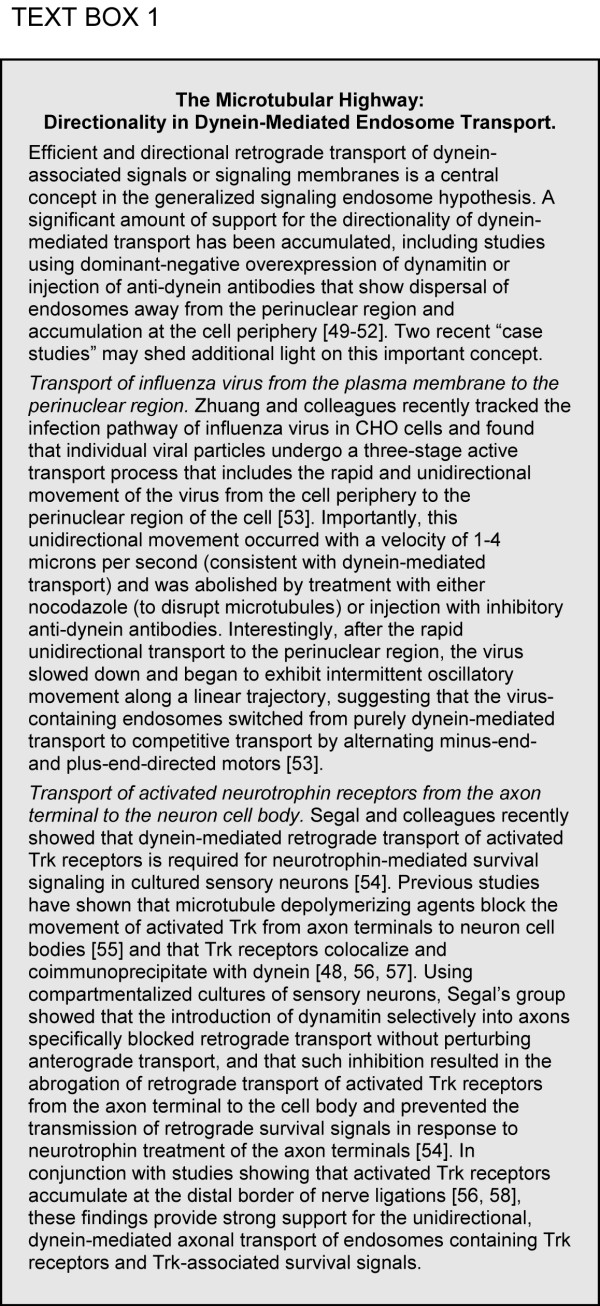
The Microtubular Highway. Evidence of the directionality of dynein-mediated retrograde transport.

We have previously provided evidence that neurotrophin-induced Erk1/2 signaling from retrogradely transported endosomes is more efficient than diffusion over distances ranging from 1.3 microns to 13 microns [7]. We also suggested that the phosphorylation signal associated with signaling endosomes is regenerative [7], consistent with our previous observations regarding the characterization of purified signaling endosomes from neurotrophin-stimulated cells [26]. Figure 4 provides additional analysis in support of the regenerative capacity of signaling endosomes. Such signal regeneration is in stark contrast to the terminal dephosphorylation experienced by diffusing signal transducers, and is a key element in favor of the signaling endosome hypothesis [4,7]. However, our previous observations depended upon the comparison of the Einstein-Stokes diffusion equation-derived root-mean-square effective distance for Erk1/2 and the average transport velocity for nerve growth factor [7]. Such a comparison overlooks a critical feature of signaling endosome transport and a critical failure of diffusion: directionality. Diffusion is inherently directionless, while the movement of signaling endosomes along microtubules is inherently directional and vectorial (see Figure 5 "The Microtubular Highway"). Likewise, simple modeling of the root-mean-square effective diffusion distance against transport velocity ignores dephosphorylation and the regenerative capacity of endosome-associated signals. Herein, we report that brute-force Monte Carlo (random walk) simulations of STAT-3 diffusion and dephosphorylation kinetics indicates that facilitated transport of endosomal-based signals is more efficient than diffusion over even very small cellular distances. Therefore, we conclude that signaling from endosomes represents a general biological principle relevant to all cell types and to all signal transduction pathways.

## Results and discussion

### Assumptions – Transport Velocity

For modeling, a dynein-based transport rate of 5 microns per second is assumed, based on a report by Kikushima and colleagues [27]. This value was used for ease of calculation: with a cell radius of 7.5 microns and a nuclear radius of 2.5 microns, a 5 μm per second transport rate moves the signaling endosome from the plasma membrane to the nucleus in one second. Actual transport rates likely range from 1–10 μm per second in cytosol or axoplasm [7].

### Assumptions – Diffusion Coefficient

The crystal structure of STAT-3B [28], deposited in the Protein Data Bank as PDB 1BG1 [29], indicates unit cell dimensions of 17.4 × 17.4 × 7.9 nm. With the caveat that this structure is bound to an 18-base nucleic acid, the volume of a STAT-3B molecule is 2400 nm^3^. Assuming a spherical molecule, STAT-3B therefore has a molecular radius of approximately 8 nm. Likewise, the molecular weight of STAT-3 is 100000 Daltons, and therefore one molecule of STAT-3 weighs 1.7 × 10^-19 ^g. The Einstein-Stokes equation for the coefficient of diffusion is:

*D *= (1/8)(*k*·*T*)/(π·γ·η)

where *k *is Boltzmann's constant, *T *is absolute temperature in degrees Kelvin, γ is the radius of the molecule, and η is the viscosity of an isotropic medium. The viscosity of axoplasm is approximately 5 centipoise [30], a value that also approximates cytoplasm [31,32]. Hence,

*k *= 1.3805 × 10^-20 ^m^2^·g·(1/(s^2^·K))

*T *= 310 K

γ = 8 × 10^-9 ^m

*m *= 1.7 × 10^-19 ^g

η = 5 g/(m·s)

Therefore, the coefficient of diffusion for a molecule of STAT-3 is:

*D *= 4.3 μm^2 ^per second

Likewise, the instantaneous velocity *v*_*x *_, the step length δ, and the step rate τ, were derived as:

*v*_*x *_ = ((*k*·*T*)/*m*)^0.5 ^= 5 m/s

δ = (1/4)(*k*·*T*)/(*v*_*x*_·π·γ·η) = 1.7 × 10^-12 ^m

τ = *v*_*x *_/δ = 2.9 × 10^12 ^sec^-1^

It is important to note that our mass estimation may substantially underestimate the actual mass of the functional STAT-3 molecular complex, described by Sehgal and colleagues as two populations with masses ranging from 200–400 kDa ("Statosome I") to 1–2 MDa ("Statosome II") [33,34]. Such a massive molecular complex certainly has important biological implications for STAT-3 diffusion. However, because no crystal structure exists for these higher molecular weight statosomes from which to calculate the molecular radius, and in order to calculate the "best-case scenario" for effective diffusion distance, we have calculated the STAT-3 diffusion coefficient on the basis of a 100 kDa monomeric molecule. The actual diffusion coefficient for STAT-3 may be 30% of the value calculated above (assuming 2 MDa mass and a four-fold increase in molecular radius to account for molecular packing of the statosome) and the root-mean-square displacement may be 50% of the value calculated below. The impact of these variables awaits further investigation.

### Assumptions – Diffusion Modeling

We modeled diffusion using a random walk algorithm in two dimensions. The choice of dimensionality was constrained by the intensive computational burden associated with three-dimensional algorithms, as discussed below (see Methods). At every iteration of the random walk two pseudo-random numbers (see Methods) were generated and used to determine the direction of movement in the x-y plane. Using the instantaneous velocity *v*_*x *_, the step length δ, and the step rate τ, defined above, we conclude that a diffusing molecule of STAT-3 will randomly walk 3 × 10^12 ^steps per second, and each step will be 1.7 × 10^-12 ^meters long. Thus, the root-mean-square displacement for STAT-3 diffusion in one second is 2.9 μm. The random walk was modeled on one second of biological time using a loop of 3 × 10^12 ^iterations. During each iteration the molecule randomly moved ± 1.7 × 10^-12 ^meters in the x-plane and ± 1.7 × 10^-12 ^meters in the y-plane.

### Assumptions – Dephosphorylation Kinetics

The decay of a phospho-protein is an exponential function mapped between the plasma membrane and the nucleus [5,35]:

α^2 ^= (*K*_*p *_)(*L*^2^/*D*)

And the probability function for dephosphorylation is:

*p*(*x*)/*p*(*m*) = (e^α*x *^– e^-α*x*^)/*x*(e^α ^– e^-α^)

Where α is a dimensionless measure of dephosphorylation probability, *K*_*p *_ is the first-order rate constant for the activity of the relevant phosphatase, *L *is the cell diameter, *D *is the diffusion coefficient, *x *is the distance from the cell center, and *m *is the distance from cell center to plasma membrane normalized to a value of one. α scales such that for α = 10, half of all phospho-molecules become dephosphorylated within approximately 0.075 units of distance from the plasma membrane to the cell center (e.g. 750 nm for a cell with 10 μm radius) [5]. In general, *K*_*p *_, the first-order rate constant of phosphatase activity, varies between 0.1 per second and 10 per second [4,35-37]. For our model *K*_*p *_ = 5 was assumed, yielding α = 8.1.

With regard to an estimate of enzymatic activity relevant to dephosphorylation of STAT-3, Todd and colleagues report a second-order rate constant of 40000/M·s for dephosphorylation of Erk1/2 [38], which gives:

*k*_*cat *_/*k*_*m *_ = 40000/M·s

Furthermore, Denu and colleagues report that diphosphosphorylated Erk1/2 peptides exhibit *k*_*m *_ values of approximately 100 μM in vitro [39]. Therefore:

*k*_*cat *_ = 4/s

Since *k*_*cat *_ measures the number of substrate molecules turned over per enzyme per second, a *k*_*cat *_ of 4 per second means that, on average, each molecule of enzyme (phosphatase) converts (dephosphorylates) 4 substrate molecules every second. Assuming a degree of molecular similarity between Erk dephosphorylation and STAT-3 dephosphorylation, and for ease of calculation, we set *k*_*cat *_ = 5 per second. It is important to note that this assumption may not be valid, but has been necessarily adopted in the absence of better biophysical data in order to illustrate the potential circumscription of diffusion by dephosphorylation.

### Assumptions – Dephosphorylation Modeling

The random walk employed for modeling STAT-3 diffusion depends upon the massively iterative generation of random numbers to describe the movement of the walking molecule in two-dimensional space. Since significant computational time was already invested in our diffusion calculations for the generation of extremely long period pseudo-random numbers, we opted to model STAT-3 dephosphorylation as a stochastic event using the following logic: for any given randomly walking molecule, the probability of encountering a phosphatase is independent of both all other molecules and all other steps in the walk. Therefore, during one second of biological time, equivalent to 3 × 10^12 ^steps in the random walk, and assuming that *k*_*cat *_ = 5 dephosphorylations per second, there will be 1.67 × 10^-12 ^dephosphorylation events per step. This can be effectively modeled as a probability test by generating a pseudo-random number on (0,1) at each step of the random walk and asking whether this number is less than 1.67 × 10^-12^. If the test is positive, the molecule is considered to be "dephosphorylated" and the random walk is truncated. High-speed modeling of time to dephosphorylation for a large number of molecules (i.e. in the absence of the random walk) led to a probability function that matched the equations described by Kholodenko [5].

### Results – Diffusion-only Model

**Figure 6:**
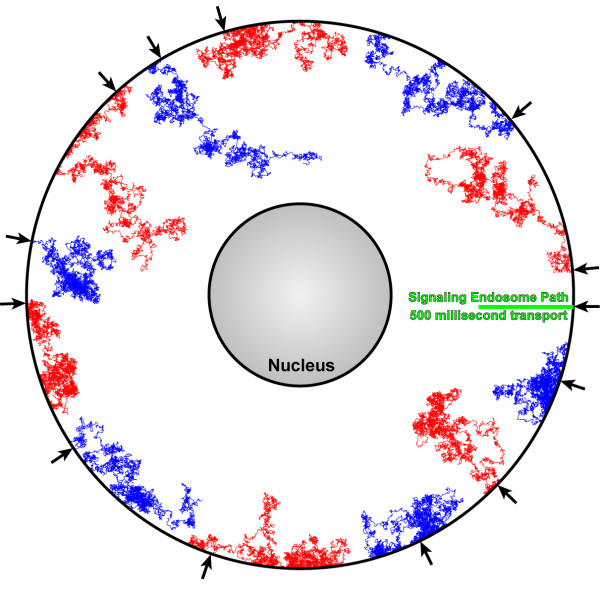
Representative trajectories for 12 random walk simulations using only diffusion criteria (red and blue lines), compared to the movement of a signaling endosome within the same 500 millisecond time frame (green line). Parameters: 15 μm cell diameter, 5 μm nucleus diameter, 37°C, 500 msec, coefficient of diffusion as described in the text. Arrows along the plasma membrane surface denote the sites of signal initiation.

Figure 6 shows the result of 12 random walks plotted in two-dimensional space and compared to the pathlength of a signaling endosome transported on microtubules. For these simulations, 500 milliseconds of biological time were modeled, resulting in the transport of the signaling endosome over 2.5 μm. The random walks were simulated using only the diffusion coefficient criteria (i.e. no dephosphorylation modeling) over the same time window. This figure illustrates the tremendous variability in the path vector for each of the diffusing particles. While not unexpected or surprising, Figure 6 offers graphic evidence that the model is working appropriately. Average pathlength analysis is discussed below.

### Results – Diffusion and Dephosphorylation Model

**Figure 7:**
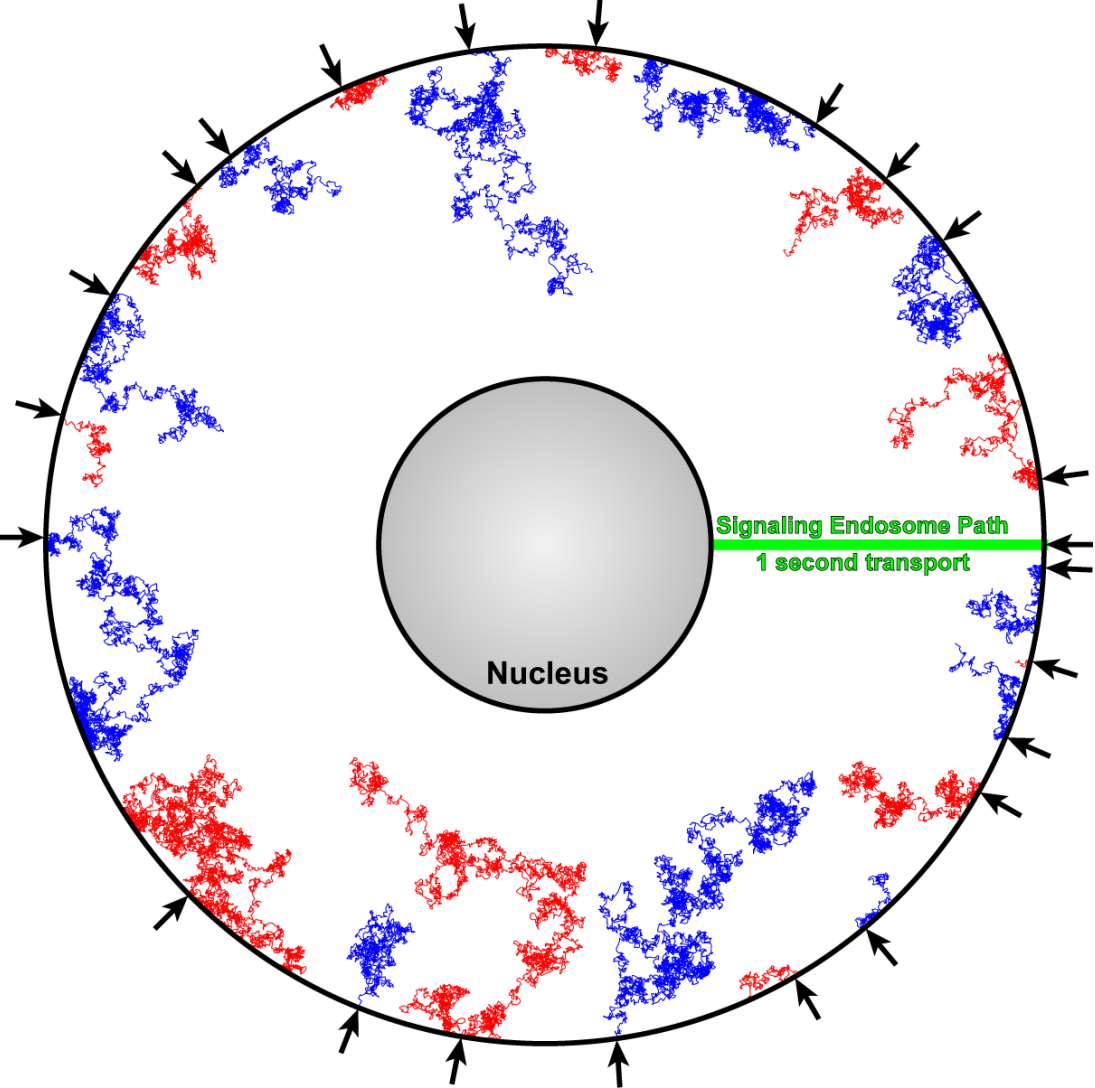
Representative trajectories for 22 random walk simulations using both diffusion and dephosphorylation criteria (red and blue lines), compared to the movement of a signaling endosome within the same 1 second time frame (green line). Parameters: 15 μm cell diameter, 5 μm nucleus diameter, 37°C, 1 sec, coefficient of diffusion and dephosphorylation probability as described in the text. Arrows along the plasma membrane surface denote the sites of signal initiation.

Figure 7 shows the result of 22 random walks modeled over one second of biological time incorporating both the diffusion coefficient criteria and the dephosphorylation probability criteria. Again, the random walks are compared to the pathlength for the transported signaling endosome, which in this case moves across the entire 5 μm distance separating the plasma membrane and the nucleus. As with Figure 6, there is a large amount of variability in the diffusion paths, but it is clear that the incorporation of dephosphorylation into the model substantially truncates the effective distance over which a diffusing molecule of STAT-3 travels. As discussed above, with α = 8.1, 50% of all phosphorylated molecules should be dephosphorylated within 0.1 distance units of the plasma membrane. For our model, this means that 50% of phospho-STAT-3 molecules should be inactivated within 750 nm of the plasma membrane (α = 8.1; x = 0.9 for p = 0.5; radius = 7.5 μm; hence x = 6.75 μm, or 750 nm from the plasma membrane). Likewise, only 15% of phosphorylated STAT-3 molecules remain active at a distance half-way between the cell center and the plasma membrane, and, assuming a nucleus of 2.5 μm radius in a cell with 7.5 μm radius, fewer than 4% of phosphorylated molecules will cross the entire distance. Our random walk incorporating the dephosphorylation probability model captures the salient features of the expected dephosphorylation kinetics.

### Results – Endpoint Analysis of Both Models

**Figure 8:**
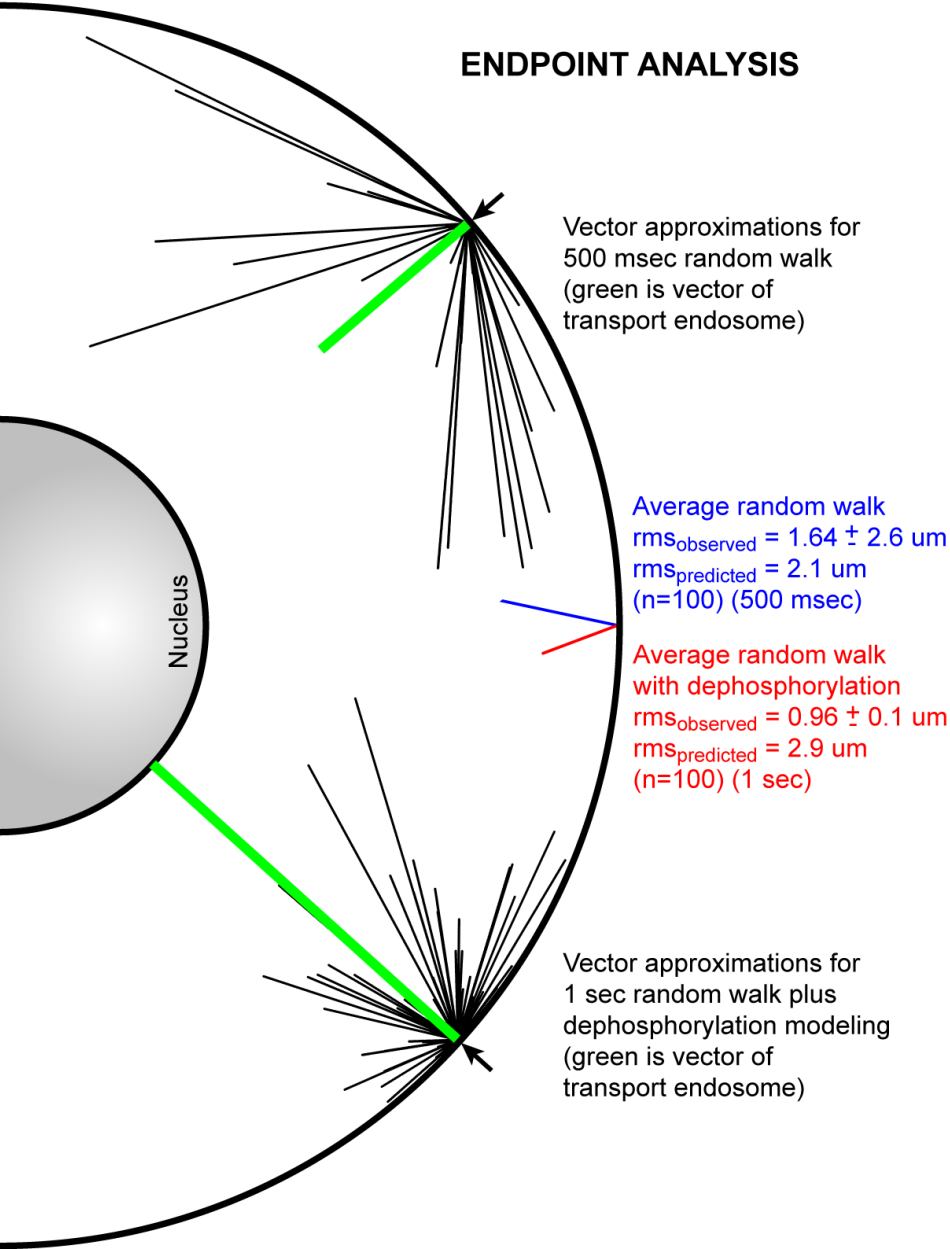
Endpoint analysis of 100 diffusion-only random walks and 100 diffusion plus dephosphorylation random walks. Black lines represent vectors calculated by the final random walk point for each simulation, compared to the distance covered by a retrogradely transported signaling endosome in the same amount of time (green lines). The blue line represents the averaged vector for 100 diffusion-only random walks, while the red line depicts the averaged vector for 100 diffusion plus dephosphorylation simulations.

Finally, Figure 8 illustrates the endpoint analysis for 100 diffusion-only random walks and 100 diffusion plus dephosphorylation walks. It should be noted that each random walk required, on average, more than 48 hours of dedicated processor time. For this analysis, the final coordinate of each diffusing molecule was used to calculate a vector for the random walk (i.e. distance and direction from point of origin). Of the 200 vectors calculated under both models, no diffusing molecule intersected the nuclear membrane within the computed timeframe. In contrast, for the one second computations incorporating both diffusion and dephosphorylation, the retrogradely transported signaling endosome reaches the nucleus with the STAT-3 phosphorylation state intact. Finally, the observed root-mean-square displacement for the 100 dephosphorylation model random walks was 0.96 μm ± 0.1 μm, or less than 20% of the distance from the plasma membrane to the nucleus. As calculated above using only the step length and step rate derived from the coefficient of diffusion parameters, the predicted root-mean-square displacement for STAT-3 is 2.9 μm. Thus, the observed effective distance for a phosphorylated STAT-3 molecule is one-third of the predicted distance, indicating that our previously published analysis substantially overestimated the range over which diffusion efficiently transmits intracellular information.

### Predictions

**Figure 9:**
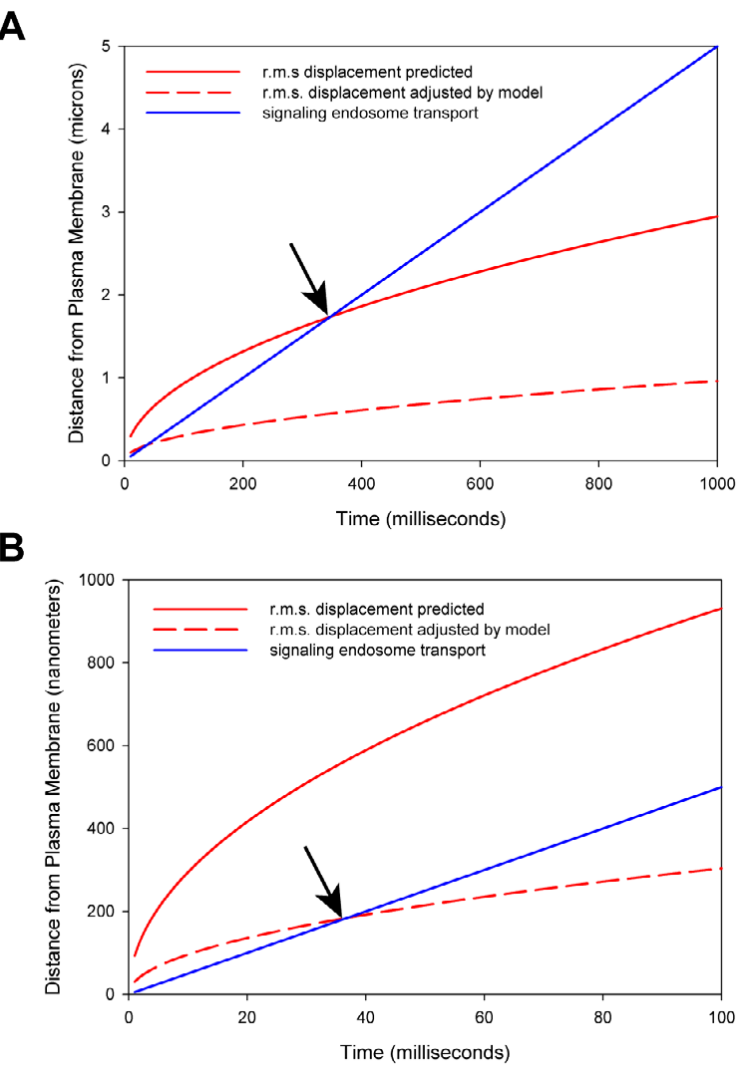
**A **and **B**) Diffusion modeling incorporating dephosphorylation kinetics indicates substantial truncation of the root-mean-square (r.m.s.) displacement for STAT-3 diffusion (dashed red line compared to solid red line). This has the effect of reducing the crossing point at which signaling endosome transport (solid blue line) overcomes diffusion (ca. 2 μm for theoretical r.m.s. vs. transport reduced to ca. 200 nm for adjusted r.m.s. vs. transport). B shows same data as A at higher Y-axis magnification.

Using the observed root-mean-square displacement after one second of biological time to establish an adjustment factor (33% of predicted), and assuming that the relationship between observed and predicted values is linear through time, we generated the plots shown in Figure 9. Figure 9A shows that the signaling endosome becomes more efficient at transmitting information from the plasma membrane over distances greater than 2 microns (greater than 400 milliseconds of biological time) using the predicted root-mean-square displacement values for comparison. However, using the adjusted root-mean-square displacement values for comparison, the signaling endosome is more efficient than diffusion within 200 nanometers from the plasma membrane (within 40 milliseconds of biological time) (Figure 9B). Therefore, our model predicts that the facilitated retrograde transport of signaling endosomes is a more efficient mechanism of information transfer from the plasma membrane to the nucleus, and is, in fact, more efficient for the transmission of phosphorylated STAT-3 signals over any distance greater than only 200 nanometers.

### Caveats and Future Directions

The signaling endosome retrograde transport rate utilized in our model may overestimate the actual transport velocity, especially as an average across the entire lifetime of the endosome-associated signal. The rate we modeled did not account for the kinetics of endocytosis or of vesicle loading onto the microtubule network. Our previous observations suggested transport velocities that ranged from 5.6 μm per second to 0.56 μm per second [7], but experiments addressing real transport rates for a variety of signaling molecules are required to improve our model. On the other hand, while we potentially overestimated the retrograde transport rate for the signaling endosome, we also very likely overestimated the size of the effective diffusion domain due to the two-dimensional restrictions of our current model. While the cytoskeletal transport of the signaling endosome is inherently a dimensionally-restricted vectorial event, diffusion within the cell most certainly occurs in three dimensions. Our current model predicts a three-fold reduction in the actual root-mean-square displacement for STAT-3 as compared to the predicted displacement using a two-dimensional random walk model, and we predict that a model incorporating three dimensions will exhibit even greater curtailment of the effective spatial domain for diffusion. However, the addition of a third dimension to the random walk simulations substantially increases computational demand, and therefore this analysis awaits either a more efficient algorithm or more computer time. Our current and future goals are to parallelize the random walk algorithm in order to perform massively parallel diffusion simulations in three dimensions.

## Conclusion

Molecular diffusion obviously benefits from the extremely high molecular velocities of single particles moving in a vacuum. For gases and other very small molecules and under conditions of low viscosity or high temperature, diffusion is extremely fast and far-ranging. However, within the context of biological molecules and biological viscosities, diffusion is vastly circumscribed [1-3,40]. Despite the limitations imposed by biological parameters, diffusion at first glance still appears to be a viable mechanism for the transmission of information through cytoplasm. In fact, the "textbook" conception of signal transduction depends upon the free diffusion of signaling molecules. However, closer scrutiny finds several faults in the diffusion model [1]. For example, diffusion is certainly directionless – even within the context of a bounded space such as the cell, the majority of molecular motions taken by a diffusing molecule are non-productive with regard to movement of signals toward a target (such as the nucleus). Likewise, a diffusing molecular signal is a ready target for interaction with and truncation by cytoplasmic phosphatases. Certainly, the effective range over which a diffusing signal maintains informational integrity depends upon the concentration and activity of equally randomly diffusing phosphatases, but it also seems likely that cells maintain levels of phosphatase sufficient to prevent run-away signal transduction [41,42]. Thus, diffusion of information is limited by both lack of direction and inevitable signal elimination. In distinct contrast, the retrograde movement of quantal signaling units capable of regenerating the information content of the original stimulus is inherently vectorial. Therefore, signaling endosomes, despite an overall lower transport velocity compared to diffusion velocities, exhibit characteristics of an optimized information transmission system. We previously sought to determine the effective range over which Erk1/2 signaling endosomes exhibited greater efficiency than diffusing Erk1/2 molecules [7]. This work relied upon the direct comparison of the root-mean-square displacement for phosphorylated Erk1/2 with the retrograde transport velocity of neurotrophin-induced signaling endosomes. In an effort to refine this model we incorporated in our present study the additional element of dephosphorylation kinetics. Thus our current model addresses both the non-vectorial nature of diffusion and the inherent susceptibility to signal truncation by interaction with cellular phosphatases. Using an iterative random walk modeling scheme we determined that the root-mean-square displacement predicted by the coefficient of diffusion for STAT-3 overestimated the root-mean-square displacement observed in our simulations by a factor of 3. Incorporating this scaling factor into the equation for root-mean-square displacement through time, we found that signaling endosomes become more effective at the transmission of information when the distance from the plasma membrane exceeds 200 nanometers. This observation suggests that any cellular situation that requires the transmission of information in the form of phosphorylated signaling molecules over distances in excess of 200 nanometers would benefit from the packaging of such signals into quantal, cytoskeleton-associated signaling packets such as signaling endosomes.

Our model suggests that cells utilize two distinct information transmission paradigms: 1) fast local signaling via diffusion over spatial domains on the order of less than 200 nanometers; 2) long-distance (>200 nanometers) signaling via information packets associated with the cytoskeletal transport apparatus. Moreover, while we have focused explicitly on the role of signaling endosomes derived from the internalization of plasma membrane receptor tyrosine kinases and associated downstream signaling partners, our model suggests that any signal that must move from the outer reaches of the cytoplasm to the perinuclear region would benefit from an association with the retrograde transport machine. For example, transcription factors may associate directly with molecular motors and chaperone proteins that protect them from dephosphorylation in a nonvesiculated manner that takes advantage of directional retrograde transport in the absence of a plasma-membrane-derived organelle. Such a mechanism was recently proposed for the transport of soluble (i.e. non-membrane-associated) activated Erk1/2 within injured axons [43]. Thus, our model supports previous observations suggesting that the signaling endosome hypothesis is a subset of a more general hypothesis that the most efficient mechanism for intracellular signaling-at-a-distance involves the association of signaling molecules with molecular motors that move along the cytoskeleton [4]. The additional benefit provided by the cytoskeletal association of membrane-bounded complexes that package a ligand-bound transmembrane receptor with downstream effector molecules is the ability to regenerate the signal at any point along the transmission path [7]. We conclude that signaling endosomes provide unique information transmission properties relevant to all cell architectures, and we propose that the majority of relevant information transmitted from the plasma membrane to the nucleus will be found in association with organelles of endocytic origin.

## Methods

### Pseudo-Random Number Generation

It should be self-evident that "built-in" pseudo-random number generators (RNGs) available in the majority of operating systems and programming languages are essentially useless for large-scale Monte Carlo simulations [44]. However, during our initial efforts to optimize the processing time for the one-second simulations we experimented with several common RNGs; all failed to exhibit sufficiently long periods, a failure that was manifested in an initial period of random walking followed by capture in a continuously repeating cyclical path. We also experimented with an implementation of the Mersenne Twister algorithm, which exhibited a robust period (theoretically 2^19937^-1) and computational demand comparable to many other standard RNGs [45]. However, our final optimized diffusion-only code utilized a multiply-with-carry RNG (MWC) described by George Marsaglia [44,46,47]. The MWC algorithm generates extremely long-period pseudo-random numbers on [0,1], and we utilized this very efficient RNG for Boolean testing of step direction in two dimensions. For the combined diffusion and dephosphorylation models, we used the Mersenne Twister modified to generate pseudo-random numbers on (0,1) for the probabilistic determination of a dephosphorylation event and the MWC algorithm for step direction determination.

### Hardware

We utilized a variety of platforms for development, testing, and implementation of the diffusion models, including the IBM Power4 p690 supercomputer (running AIX 5.2) and the SGI Altix 3700 supercomputer (running SGI Advanced Linux 3.4) at the University of Minnesota Supercomputing Institute. The serial models described above were primarily implemented on a single processor Intel P4 3.0 GHz machine running Red Hat Linux 9.0. The IBM Power4, the SGI Altix 3700, and a dual processor Xeon 3.0 GHz Nocona box running Red Hat Enterprise Linux 3.0 were used for development and testing of parallel implementations. Total wallclock time on all platforms currently exceeds 10000 hours.

### Software

All algorithms were coded in C and compiled with gcc or xlc (serial implementations) or with pgcc, xlc, or icc (OpenMP parallel implementations). Our first diffusion model efforts required more than one week of dedicated processing time per walk; after several rounds of code optimization we could obtain one second of simulated time in approximately 48 hours on the Power4 architecture and the Pentium 4 architecture described above.

## Competing interests

The author(s) declare that they have no competing interests.

## Authors' contributions

The author contributed to all phases of the work.
